# Spinal Cord Injury-Related Male Infertility: Oxidative Stress, Inflammation, and Sperm DNA Damage

**DOI:** 10.3390/jcm15166312

**Published:** 2026-08-15

**Authors:** Athanasios Zikopoulos, Athanasios Zachariou, Maria Filiponi, Grigorios Daligkaros, Alexandros Zikopoulos, Dimitrios Zachariou, Vasilios-Sebastian Paraschos, Sofia Markoula

**Affiliations:** 1 University College London Hospital, London NW1 2BU, UK; 2 Department of Urology, University of Ioannina, 45500 Ioannina, Greece; 3 Physical Medicine and Rehabilitation Centre Kentavros, 38222 Volos, Greece; 4 1st Department of Orthopedic Surgery, ATTIKO University General Hospital, 12462 Athens, Greece; 5 Department of Obstetrics and Gynecology, Corewell Health Hospital, 4700 Schaefer Road, Suite 310, Dearborn, MI 48126, USA; 6 Department of Neurology, University of Ioannina, 45110 Ioannina, Greece

**Keywords:** spinal cord injury, male infertility, oxidative stress, reactive oxygen species, inflammation, sperm DNA fragmentation, blood–testis barrier, cytokines, assisted reproduction

## Abstract

Spinal cord injury is strongly associated with male infertility, traditionally attributed to erectile and ejaculatory dysfunction. However, accumulating evidence demonstrates that infertility in this population extends beyond neurogenic impairment and involves profound biochemical and molecular alterations within the male reproductive system. The objective of this review is to synthesize current evidence on the role of oxidative stress, inflammation, and sperm DNA damage as central mechanisms driving impaired fertility in men with spinal cord injury. A comprehensive evaluation of clinical and experimental studies indicates that seminal plasma in affected individuals is characterized by elevated reactive oxygen species, reduced antioxidant capacity, and a pro-inflammatory cytokine profile, including increased levels of tumor necrosis factor alpha, interleukin 6, and interleukin 1 beta. These alterations are closely linked to leukocytospermia, mitochondrial dysfunction, and disruption of the blood–testis barrier, creating a toxic microenvironment that compromises sperm function. As a consequence, spermatozoa exhibit reduced motility, impaired membrane integrity, and significantly increased DNA fragmentation and chromatin abnormalities. Elevated sperm DNA damage has been consistently associated with poor fertilization outcomes, reduced embryo quality, and increased risk of pregnancy loss in assisted reproductive settings. Importantly, these molecular defects persist even when sperm are retrieved using advanced techniques such as electroejaculation or surgical extraction, highlighting the need for targeted therapeutic strategies. Interventions aimed at reducing oxidative stress and modulating inflammation, including antioxidant supplementation and advanced sperm selection methods, show promise but remain insufficiently standardized. Future research should focus on the development of personalized approaches integrating molecular diagnostics and targeted therapies to improve reproductive outcomes in this population. Collectively, this review reframes spinal cord injury-related infertility as a complex inflammatory and oxidative disorder and underscores the importance of addressing sperm DNA integrity in clinical management.

## 1. Introduction

The consequences of spinal cord injury (SCI) on the male reproductive system represent one of the most complex challenges in modern andrology and rehabilitative medicine [1]. While historical clinical focus was understandably directed toward the restoration of motor function and bladder management, the demographic profile of SCI patients, predominantly young men in their peak reproductive years, has shifted the priority toward the preservation of fertility [1,2]. The peak incidence of traumatic SCI occurs between the second and fourth decades of life, precisely the period in which family planning is most relevant, and improved acute and long-term survival now means that reproductive counseling has become an integral component of comprehensive rehabilitation. It is a common clinical misconception that infertility in this population is solely a byproduct of neurogenic mechanical failure. While it is true that the majority of men with SCI face significant hurdles regarding erectile and ejaculatory function, the physiological reality is far more insidious. Even when viable spermatozoa are retrieved, the success rates of both natural and assisted conception remain disappointingly low, pointing to a profound and progressive degradation of sperm quality that begins almost immediately following the initial trauma [3].

The pathophysiology of this decline is not rooted in a single anatomical defect but rather in a systemic collapse of the testicular and post-testicular microenvironments. In the acute and chronic phases of spinal cord injury, the reproductive tract is subjected to a “perfect storm” of biological stressors. These include the loss of autonomic thermoregulation, which leads to chronic scrotal hyperthermia, venous stasis within the pampiniform plexus, and a state of persistent immune activation. This inflammatory milieu is characterized by a significant influx of activated leukocytes into the seminal plasma, a condition known as leukocytospermia. The presence of these cells, combined with the stasis of sperm in the efferent ducts due to infrequent ejaculation, triggers a cascade of oxidative stress that fundamentally alters the molecular integrity of the male gamete [1,4].

Central to this biochemical disruption is the overwhelming production of reactive oxygen species (ROS) which outpaces the body’s endogenous antioxidant defenses. In the context of SCI, this oxidative imbalance does not merely exist as a bystander effect; it is the primary driver of mitochondrial dysfunction and subsequent sperm DNA fragmentation [5]. Because the sperm plasma membrane is exceptionally rich in polyunsaturated fatty acids, it is uniquely vulnerable to lipid peroxidation induced by ROS. This damage compromises the fluidity of the membrane and the stability of the axoneme, leading to the hallmark clinical presentation of SCI-related infertility: near-normal sperm concentrations paired with a catastrophic loss of motility and viability [6]. This dissociation between quantity and quality distinguishes SCI-related infertility from most other forms of male subfertility and explains why conventional semen parameters alone poorly predict reproductive potential in this group.

Furthermore, the disruption of the blood–testis barrier (BTB) in the wake of the initial spinal trauma allows for the potential development of anti-sperm antibodies, further complicating the immunological landscape of the reproductive tract [7]. This review seeks to move beyond the traditional focus on neurogenic dysfunction to explore these sophisticated molecular mechanisms. By synthesizing the current evidence on cytokine signaling, oxidative pathways, and genomic instability, we aim to provide a comprehensive framework for understanding why the SCI seminal environment becomes so toxic to sperm health and how targeted therapeutic interventions might eventually reverse these “invisible” barriers to paternity.

## 2. Methods

A comprehensive literature search was conducted across three electronic databases—MEDLINE (via PubMed), Web of Science, and Scopus—to identify relevant publications from the past two decades. The search strategy incorporated both controlled vocabulary and free-text terms related to spinal cord injury, male infertility, sperm function, oxidative stress, inflammation, and therapeutic management. To maximize the retrieval of pertinent evidence, the reference lists of included articles and other influential publications were manually screened for additional studies.

Studies were considered eligible if they involved adult men with spinal cord injury and addressed infertility or reproductive outcomes. Priority was given to investigations that clearly described treatment strategies and intervention protocols. Eligible study designs included randomized controlled trials, as well as prospective and retrospective cohort studies. To ensure consistency in data extraction and interpretation, only articles published in English were included.

**Figure 1:**
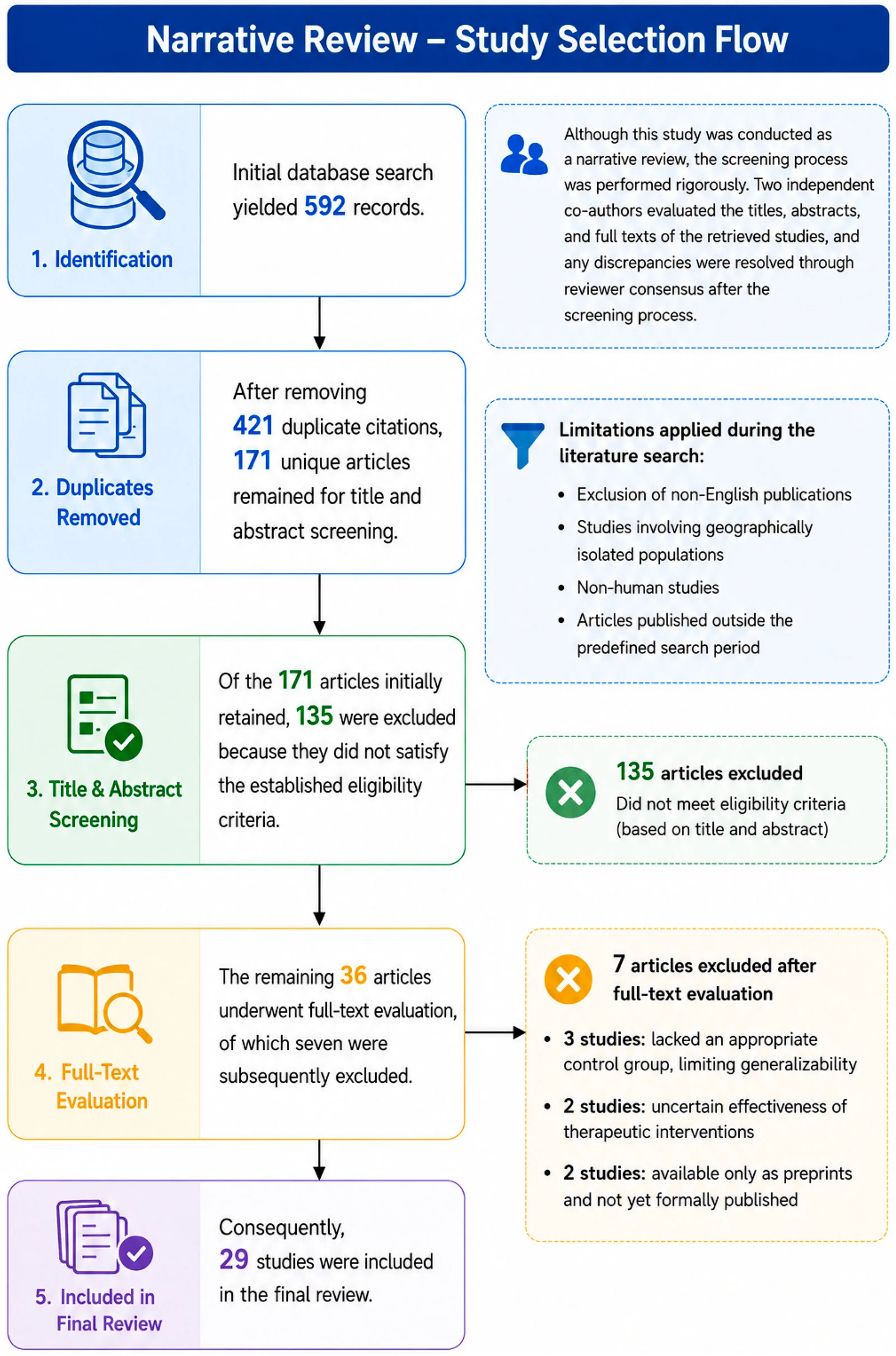
Flow diagram of the study selection process for the narrative review.

The initial database search yielded 592 records. After removing 421 duplicate citations, 171 unique articles remained for title and abstract screening. Although this study was conducted as a narrative review, the screening process was performed rigorously. Two independent co-authors evaluated the titles, abstracts, and full texts of the retrieved studies, and any discrepancies were resolved through reviewer consensus after the screening process. Several limitations were applied during the literature search, including the exclusion of non-English publications, studies involving geographically isolated populations, non-human studies, and articles published outside the predefined search period. Of the 171 articles initially retained, 135 were excluded because they did not satisfy the established eligibility criteria. Consequently, 36 studies met the inclusion criteria based on their titles, abstracts, and full-text assessments and were included in the final review. The remaining 36 articles underwent full-text evaluation, of which seven were subsequently excluded. More specifically, three studies were excluded because they lacked an appropriate control group, limiting the generalizability of their findings. Two additional studies were omitted due to uncertainty regarding the effectiveness of the therapeutic interventions they evaluated. Furthermore, two studies were excluded because they were available only as preprints and had not yet undergone formal peer-reviewed publication, making them preliminary sources of evidence. Consequently, 29 studies were included in the final review (Figure 1).

The methodological quality of the studies included in this narrative review was assessed using a Level of Evidence (LoE) framework, which categorizes research according to the robustness of its design and the reliability of the evidence it provides. Each study was independently evaluated by two reviewers and assigned an appropriate evidence level based on its methodological characteristics. Any discrepancies in classification were discussed until consensus was reached. Studies with the strongest methodological designs, such as randomized controlled trials and systematic reviews, were assigned the highest evidence levels, whereas non-randomized controlled trials, cohort studies, case–control studies, case series, and expert opinion or preliminary reports were ranked at progressively lower levels. Applying this structured hierarchy enabled a consistent evaluation of the methodological strength of the available literature and facilitated comparisons among studies. The assessment showed that most of the included studies (24 of 29) were supported by moderate-quality evidence, while the remaining studies were supported by lower-quality evidence due to inherent methodological limitations. Incorporating the LoE framework enhanced the rigor and transparency of the review by enabling the findings to be interpreted within the context of the overall strength of the available scientific evidence.

A narrative review methodology was selected to synthesize the available evidence and provide a comprehensive overview of the relationship between spinal cord injury and male infertility. Unlike systematic reviews, which employ rigid methodological frameworks and predefined selection procedures, narrative reviews allow for a more flexible integration of diverse sources of evidence, including clinical studies, mechanistic research, theoretical perspectives, and expert opinion. This approach is particularly appropriate for topics characterized by heterogeneous evidence and complex biological mechanisms, enabling a broader discussion of the multifactorial pathways linking spinal cord injury with impaired male reproductive function and the current strategies for its clinical management.

## 3. Neurogenic Barriers to Fertility: Erectile and Ejaculatory Dysfunction

The mechanical foundations of male fertility, erection, emission, and expulsion, are governed by a precise synergy between the sympathetic, parasympathetic, and somatic nervous systems. In the context of spinal cord injury, the interruption of descending supraspinal pathways and the potential damage to localized spinal centers create a profound disconnection between sexual stimulus and physiological response [1,3]. The extent of this impairment is rarely uniform; rather, it is dictated by the neurological level of the lesion and the degree of spinal cord sparing. While the loss of motor and sensory function is the most visible consequence of SCI, the disruption of the autonomic “cross-talk” required for coordinated reproduction remains a primary barrier to natural conception in this population [8].

### 3.1. The Neuro-Anatomical Basis of Erectile Failure

Erectile capacity post-injury is traditionally categorized by the preservation of either psychogenic or reflexogenic pathways. Psychogenic erections, which are triggered by auditory, visual, or imaginative stimuli, require intact descending pathways from the brain to the thoracolumbar sympathetic outflow (T11 through L2). Consequently, men with complete lesions above the T11 level typically lose the ability to achieve psychogenic erections, as the brain’s signals cannot bypass the site of trauma [9].

In contrast, reflexogenic erections are mediated by the sacral parasympathetic centers located at S2 to S4. These erections occur in response to direct physical stimulation of the genital region and can often be maintained in patients with cervical or high-thoracic injuries, provided the sacral reflex arc remains functional. The clinical corollary is predictable from the neuro-anatomy: reflexogenic capacity is preserved in the large majority of men with upper motor neuron lesions but is frequently lost when the sacral cord or its roots are directly involved. However, the quality of these erections is frequently suboptimal for penetrative intercourse, characterized by insufficient rigidity or an inability to sustain the tumescence long enough for successful coitus. This instability in erectile function serves as the initial, mechanical hurdle in the reproductive journey of the SCI patient [10].

### 3.2. Ejaculatory Dysfunction and the Mechanism of Anejaculation

While erectile dysfunction is a significant concern, the near-total prevalence of anejaculation (estimated in some cohorts to exceed 90%) represents the most formidable neurogenic obstacle to fertility. Ejaculation is a two-phase process: emission and expulsion. Emission involves the contraction of the vasa deferentia, seminal vesicles, and prostate to deliver semen into the posterior urethra, a process primarily controlled by sympathetic fibers arising from the T10 to L2 segments. Expulsion follows as a rhythmic, somatic reflex involving the bulbocavernosus and pelvic floor muscles, coordinated through the pudendal nerve and the sacral spinal cord [11].

Spinal cord injury typically severs the coordination between these two phases. In many patients, the sympathetic trigger for emission is either absent or dyssynchronic, leading to a failure of semen to enter the urethra. Furthermore, even when emission occurs, the internal urethral sphincter, which should close to prevent semen from entering the bladder, often remains open or fails to coordinate with the pelvic floor contractions. This results in retrograde ejaculation, where the ejaculate is diverted into the bladder, further complicating any attempts at home-based insemination. The loss of this “ejaculatory reflex” necessitates the medical interventions described later in this review, shifting the burden of reproduction from natural processes to clinical sperm retrieval [1,12,13].

## 4. Oxidative Stress in Spinal Cord Injury

A defining characteristic of infertility in the spinal cord injury population is the paradoxical preservation of sperm concentration alongside a catastrophic decline in progressive motility and membrane integrity. This dissociation suggests that the primary insult is not an arrest of spermatogenesis *per se*, but rather a profound post-testicular degradation of the gamete mediated by a hostile seminal microenvironment [4]. Central to this degradation is the accumulation of reactive oxygen species (ROS) and a systemic shift toward a pro-inflammatory cytokine profile within the accessory sex glands and the efferent ductal system [14].

### 4.1. Sources of Reactive Oxygen Species and Redox Imbalance

The spermatozoa of men with SCI are subjected to an overwhelming oxidative burden that originates from both endogenous and exogenous sources. Endogenously, the stasis of sperm within the seminal vesicles, a consequence of infrequent ejaculatory cycles, leads to the accumulation of aged and apoptotic cells which naturally leak electrons from the mitochondrial transport chain [6,15,16]. These electrons reduce molecular oxygen to superoxide anions (O_2_^−^), initiating a cascade of oxidative damage that is amplified by the parallel decline in seminal total antioxidant capacity. Exogenously, the prevalence of chronic asymptomatic bacteriuria and recurrent urinary tract infections, a near-universal consequence of neurogenic bladder and intermittent catheterization, recruits activated polymorphonuclear leukocytes into the semen, a condition termed leukocytospermia [17].

### 4.2. Mitochondrial Dysfunction and Lipid Peroxidation

These leukocytes are potent producers of hydrogen peroxide (H_2_O_2_) and hydroxyl radicals, which target the sperm plasma membrane. Because the mammalian sperm membrane is uniquely enriched with polyunsaturated fatty acids to facilitate the fluidity required for fertilization, it is exceptionally susceptible to lipid peroxidation. This process creates a self-propagating cycle of membrane damage, leading to the formation of cytotoxic aldehydes such as malondialdehyde and 4-hydroxynonenal and the eventual loss of axonemal structural integrity. Furthermore, this oxidative stress induces a significant reduction in the mitochondrial membrane potential (MMP), effectively starving the sperm of the ATP required for flagellar propulsion and acrosomal activation [6,18]. The resulting collapse of energy metabolism, coupled with membrane rigidity, provides a coherent mechanistic explanation for the severe asthenozoospermia that characterizes SCI ejaculates despite preserved sperm counts and it establishes mitochondrial injury as an early and pivotal event that precedes DNA damage.

## 5. Inflammation, Cytokines and the Blood–Testis Barrier

The immunological landscape of the SCI reproductive tract is characterized by a chronic elevation in pro-inflammatory signaling molecules, most notably tumor necrosis factor-alpha (TNF-a) and various interleukins such as IL-1β, IL-6, and IL-10. These cytokines do not merely serve as markers of inflammation but act as bioactive agents that modulate the permeability of the blood–testis barrier (BTB). Under physiological conditions, the BTB, formed by tight junctions between adjacent Sertoli cells, maintains an immunologically privileged site within the seminiferous tubules, protecting developing germ cells from systemic immune recognition [19,20,21]. Oxidative stress and inflammation are mechanistically intertwined rather than independent: ROS activate redox-sensitive transcription factors such as NF-κB, which in turn drive further cytokine production, establishing a self-reinforcing feed-forward loop.

However, the persistent inflammatory state observed post-SCI, exacerbated by neurogenic bladder complications and the frequent use of intermittent catheterization, appears to compromise the tight junctions of the BTB. This “leakage” facilitates the entry of inflammatory mediators into the testicular parenchyma and potentially allows for the exposure of sequestered germ cell antigens to the systemic circulation. Such exposure may trigger the production of anti-sperm antibodies (ASAs), which further impair fertility by inducing sperm agglutination or inhibiting the essential sperm–oocyte interaction. Consequently, the SCI seminal environment represents a complex interplay of redox instability and immunological failure, where the synergy of these forces creates a milieu that is fundamentally incompatible with normal sperm function [7,22,23]. A simplified summary of commonly reported findings is presented below in Table 1.

## 6. Sperm DNA Damage, Apoptosis, and Epigenetic Consequences

**Figure 2:**
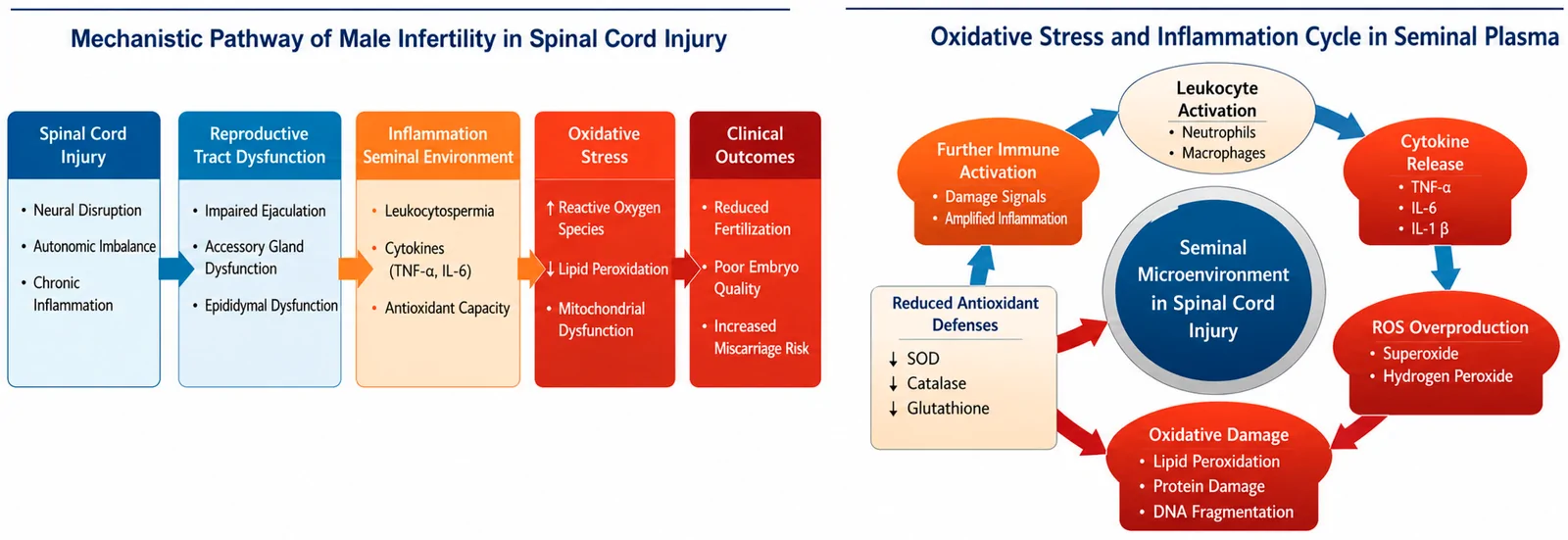
Mechanistic pathway of male infertility in SCI.

The culmination of the oxidative and inflammatory stressors present in the spinal cord injury microenvironment is the physical degradation of the paternal genome. While traditional semen analysis often focuses on the external characteristics of the sperm such as morphology and motility, the integrity of the condensed chromatin within the sperm head is perhaps the most critical determinant of successful embryogenesis and healthy live birth rates [4,24,25]. The mechanistic pathway that links the hostile seminal environment to impaired reproductive outcomes is summarized in Figure 2.

### 6.1. Molecular Mechanisms of Oxidative DNA Damage

In the context of spinal cord injury, sperm DNA fragmentation occurs at significantly higher frequencies compared to the broader fertile population, with many cohorts reporting DNA fragmentation indices well above the thresholds generally regarded as clinically detrimental. This genomic instability is primarily a post-testicular event, arising as the spermatozoa traverse the epididymis and mix with the highly reactive secretions of the accessory glands. The molecular mechanism of this damage is largely attributed to the induction of single- and double-stranded DNA breaks through the heavy burden of reactive oxygen species, with the guanine base being especially vulnerable to oxidation to 8-hydroxy-2′-deoxyguanosine, a widely used molecular marker of oxidative DNA injury. When these oxygen radicals surpass the limited cytosolic antioxidant capacity of the sperm, they initiate a process of base modification and phosphate backbone cleavage [6]. Unlike somatic cells, mature spermatozoa have shed the majority of their cytoplasm and possess almost no DNA repair machinery, meaning that any damage sustained during transit through the male reproductive tract is permanent. This damage can only be potentially addressed by the oocyte machinery after fertilization has already occurred. If the oocyte repair capacity is overwhelmed by the extent of the paternal DNA lesions, the clinical result is typically characterized by poor blastocyst development, failed implantation, or recurrent early pregnancy loss [26,27].

### 6.2. Apoptotic Signaling and Programmed Cell Death

Furthermore, the high level of DNA fragmentation in these patients is closely associated with an advanced state of sperm apoptosis. Although these cells may appear viable under a light microscope, they often display externalized phosphatidylserine and activated caspases, which are biochemical hallmarks of programmed cell death, together with the retention of excess residual cytoplasm that further fuels ROS generation. The persistence of these apoptotic markers indicates that the sperm are undergoing a silent degradation that standard concentration and motility parameters fail to capture [28]. This genomic decay explains why many men with spinal cord injury fail to achieve a pregnancy even when using advanced assisted reproductive technologies such as intracytoplasmic sperm injection. The focus of clinical assessment must therefore shift toward the molecular quantification of DNA integrity, utilizing assays such as the sperm chromatin structure assay or the terminal deoxynucleotidyl transferase dUTP nick end labeling method to accurately evaluate the reproductive potential of this specific patient cohort [29,30,31].

### 6.3. Epigenetic Alterations and Developmental Consequences

The epigenetic implications of this damage also warrant significant concern, as oxidative stress can alter the methylation patterns of the paternal chromatin. These epigenetic shifts may have long-term consequences for the health of the offspring, suggesting that the impact of spinal cord injury on reproduction is not limited to the act of conception but extends to the developmental trajectory of the next generation. Because sperm DNA serves as the blueprint for the early embryo, any disruption in the protamine transition or the methylation status of imprinted genes can lead to neurodevelopmental or metabolic abnormalities in the progeny [32,33,34]. This adds a layer of complexity to the counseling of spinal cord injury patients, as the goal of therapy must transition from simply achieving a pregnancy to ensuring the genomic and epigenetic health of the future child [35]. A summary of the clinical associations between DNA sperm damage, chromatin integrity, and reproductive outcomes is presented in Table 2.

## 7. Seminal Plasma as an Active Mediator of Infertility

Although much attention has been directed toward intrinsic sperm defects, the surrounding seminal plasma plays an equally critical role in shaping sperm function. In men with spinal cord injury, seminal plasma is not simply altered in composition but appears to act as a biologically active medium that actively impairs sperm performance. This shift transforms the ejaculate from a supportive environment into one that is functionally hostile [29,36].

One of the most consistent observations is that spermatozoa from healthy donors exhibit reduced motility and viability when exposed to seminal plasma from men with spinal cord injury. Conversely, sperm from affected individuals may demonstrate partial functional recovery when placed in a normal seminal or culture environment. These reciprocal mixing experiments strongly suggest that the seminal fluid itself contains factors that directly contribute to sperm dysfunction [4,37]. Several mechanisms appear to underlie this phenomenon. First, the balance between oxidants and antioxidants within seminal plasma is profoundly disrupted. As described earlier, elevated reactive oxygen species combined with reduced antioxidant capacity create a sustained oxidative burden. This environment not only damages sperm membranes but also affects key processes such as capacitation and the acrosome reaction, which are essential for fertilization [6,38].

In parallel, inflammatory mediators accumulate within the seminal compartment. Increased levels of cytokines and chemokines alter the biochemical landscape, influencing sperm metabolism and survival. These molecules can interfere with membrane signaling pathways, disrupt mitochondrial function, and promote apoptotic changes, and their effects are often synergistic with oxidative stress, further amplifying cellular damage. Changes in the physical and biochemical properties of seminal plasma also contribute to its detrimental role. Alterations in pH and osmolarity can affect sperm motility and membrane stability [39]. In addition, variations in protein composition, including enzymes and binding proteins derived from accessory glands, may impair sperm maturation and function. Proteomic analyses have revealed differences in proteins related to immune regulation, oxidative balance, and cellular stress responses, suggesting a broad reprogramming of the seminal environment [40,41].

Emerging evidence also points toward a role for extracellular vesicles, including exosomes and prostasomes, in mediating these effects. Under physiological conditions, these vesicles facilitate communication between cells and contribute to sperm maturation within the epididymis and to the modulation of motility and capacitation. In the context of spinal cord injury, however, their content may be altered, carrying inflammatory signals or oxidative stress-related molecules that negatively influence spermatozoa [42]. The central importance of seminal plasma is further supported by clinical observations in assisted reproduction. Techniques that separate sperm from seminal fluid, such as density gradient centrifugation or swim-up methods, often result in improved sperm function. However, these approaches do not fully reverse the underlying damage, particularly at the level of DNA integrity, indicating that exposure to the altered seminal environment may have already exerted irreversible effects [43].

**Figure 3:**
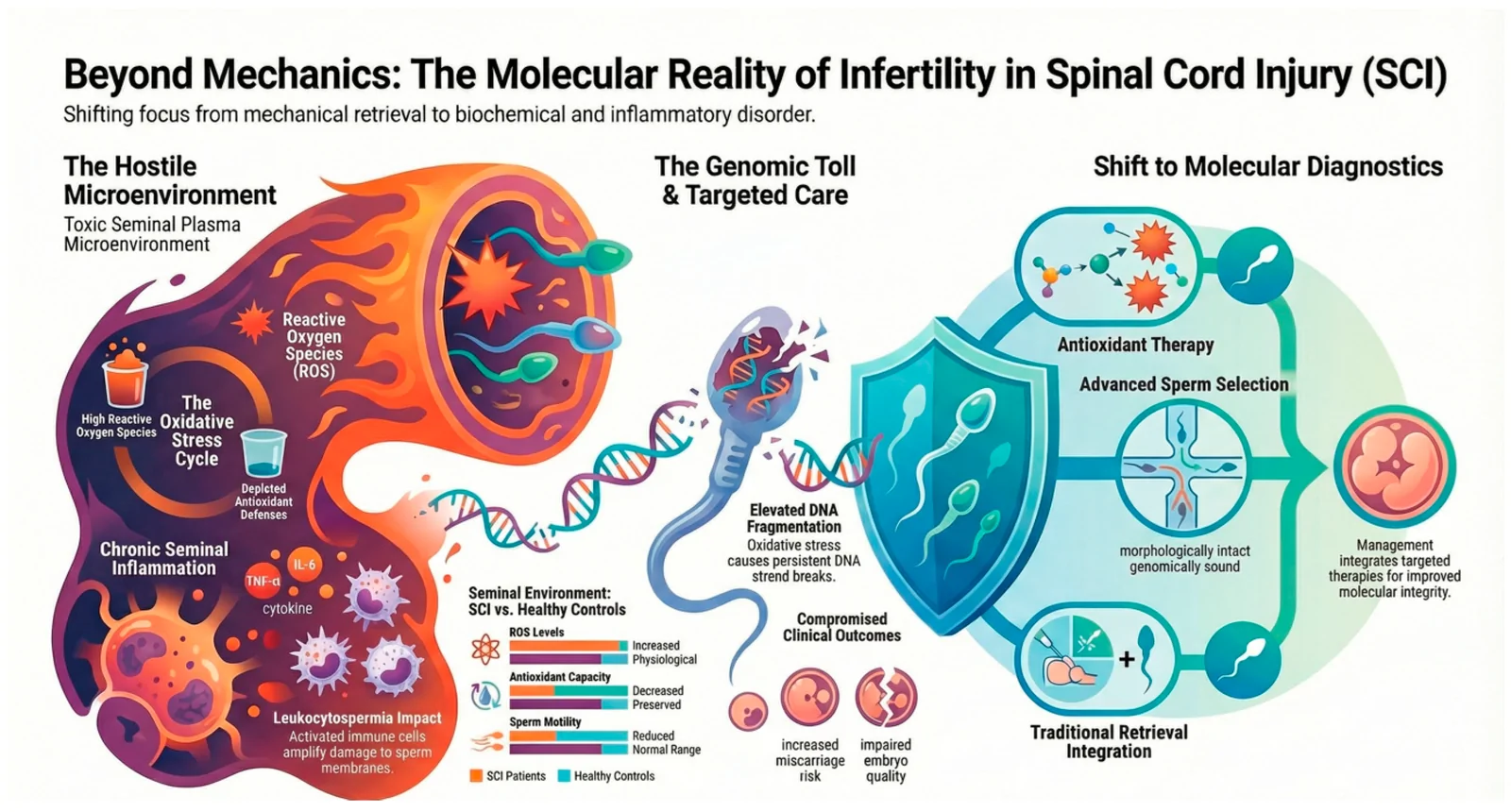
SCI creates a hostile seminal microenvironment characterized by chronic inflammation, leukocytospermia, increased cytokines, and elevated reactive oxygen species combined with reduced antioxidant defenses. This imbalance drives oxidative stress and results in sperm DNA fragmentation, impaired motility, and compromised reproductive outcomes, including reduced embryo quality and increased miscarriage risk. The figure also illustrates the shift from mechanical sperm retrieval toward molecular diagnostics and targeted interventions, such as antioxidant therapy and advanced sperm selection, aiming to improve sperm genomic integrity and overall fertility outcomes.

These findings position seminal plasma as a key mediator in spinal cord injury-related infertility. Rather than serving a purely supportive role, it becomes an active participant in the pathological process, integrating oxidative stress, inflammation, and biochemical alterations into a single functional outcome. This perspective helps bridge the gap between systemic changes and local sperm dysfunction, and provides a rationale for targeted interventions aimed at modifying the seminal environment as illustrated in Figure 3.

### Critical Appraisal of the Available Evidence

Although the available literature consistently supports a central role for oxidative stress, inflammation, and sperm DNA damage in spinal cord injury-related male infertility, several methodological limitations should be considered when interpreting these findings. First, many of the included studies were based on relatively small patient cohorts, reflecting the limited availability of well-characterized SCI populations and reducing the statistical power and generalizability of the reported outcomes. Second, considerable heterogeneity exists across studies with respect to injury level and completeness, duration of injury, semen collection methods, laboratory assays used to quantify oxidative stress and DNA fragmentation, and therapeutic interventions evaluated. This variability limits direct comparison between studies and precludes firm conclusions regarding the efficacy of specific treatment strategies. Furthermore, most studies employed observational or non-randomized designs, making them susceptible to selection bias, confounding, and incomplete adjustment for important clinical variables, such as urinary tract infections, bladder management, medication use, and lifestyle factors, all of which may independently influence semen quality. Moreover, although oxidative stress and inflammation are consistently implicated in SCI-associated infertility, several mechanistic pathways—including blood–testis barrier disruption, mitochondrial dysfunction, and cytokine-mediated toxicity—remain incompletely established in humans, with much of the mechanistic evidence derived from experimental or animal models. While the overall body of evidence provides biologically plausible and clinically consistent findings, there remains a need for larger, multicenter prospective studies and well-designed randomized controlled trials using standardized outcome measures to strengthen the evidence base and facilitate translation into clinical practice.

## 8. Clinical Management

The clinical management of infertility in men with spinal cord injury requires a multifaceted approach that addresses the neurological barriers to sperm delivery and the biochemical barriers to sperm quality. Because the majority of these patients are unable to achieve natural conception through intercourse, the primary goal of initial intervention is the reliable retrieval of a high-quality semen sample. This process is complicated by the wide variability in lesion levels and the degree of autonomic sparing among the patient population [30,44].

### 8.1. Management of Erectile Dysfunction

The treatment of erectile dysfunction (ED) in individuals with SCI generally follows the same therapeutic principles recommended for the broader population adapted to the specific functional limitations of each patient. Pharmacological management is centered on phosphodiesterase type 5 inhibitors (PDE5is), including sildenafil, tadalafil, vardenafil, and avanafil, which are considered first-line therapy. Clinical studies have consistently demonstrated that these agents are both safe and effective in improving erectile function in men with SCI, provided there are no contraindications such as concurrent nitrate use; their efficacy is greatest when the sacral reflex arc is at least partially reserved [45,46].

When oral therapy is ineffective or contraindicated, alternative treatments include vacuum erection devices, intracavernosal injections, intraurethral alprostadil, and penile prostheses [47]. Vacuum devices can provide satisfactory erections but require caution in men with impaired penile sensation because of the risk of skin injury. Intracavernosal injection therapy is an effective second-line option for patients who do not respond to phosphodiesterase type 5 inhibitors, whereas penile prosthesis implantation remains a reliable solution for refractory cases. The choice of prosthesis should be individualized according to the patient’s functional abilities, with malleable implants often preferred in men with tetraplegia because of reduced hand dexterity [48,49]. Although these interventions restore erectile function, they do not address the underlying impairment in sperm quality, which remains the principal focus of this review.

### 8.2. Penile Vibratory Stimulation and Electroejaculation

For patients with injuries above the tenth thoracic vertebrae in whom the sacral reflex arc is intact, penile vibratory stimulation is considered the first-line, non-invasive treatment. This technique utilizes the intact reflex arc to induce a coordinated ejaculatory response and is generally performed with a high-amplitude vibrator applied to the dorsal glans. It is often preferred because it can be performed in the clinic or, in selected couples, at home, and because it tends to yield samples with higher motility and lower concentrations of inflammatory markers compared to more invasive methods [50]. A recognized hazard of penile vibratory stimulation, and of ejaculation more generally, in men with lesions at or above T6 is autonomic dysreflexia, a potentially dangerous surge in blood pressure that requires monitoring and, when necessary, pharmacological prophylaxis.

When vibratory stimulation is unsuccessful, or when the reflex arc is not intact, electroejaculation serves as a robust alternative. This procedure involves the direct electrical stimulation of the pelvic sympathetic nerves through a transrectal probe to induce seminal emission. While electroejaculation is highly successful in terms of procurement across a wide range of lesion levels, the resulting samples often exhibit poor quality due to the thermal and electrical exposure required and the potential for retrograde flow into the bladder. In such cases, the bladder must be catheterized and prewashed with a buffered medium to protect the recovered spermatozoa from the acidic and osmotic damage of urine [51,52].

### 8.3. Surgical Sperm Retrieval

In cases where non-invasive and neurogenic ejaculatory methods fail, or where the semen quality remains profoundly compromised, surgical sperm retrieval becomes necessary. Techniques such as testicular sperm extraction (TESE) or microsurgical epididymal sperm aspiration (MESA) allow for the collection of gametes that have not yet been exposed to the inflammatory environment of the accessory sex glands and the prolonged post-testicular oxidative transit. These surgically retrieved sperm are almost exclusively used in conjunction with intracytoplasmic sperm injection. While these advanced methods bypass the need for high motility or natural penetration, the success of the procedure still depends heavily on the underlying genomic integrity of the sperm; importantly, they do not eliminate injury that may already be present at the level of spermatogenesis. There is nonetheless a mechanistic and increasingly clinical rationale for the observation that testicular sperm may exhibit lower DNA fragmentation than ejaculated samples in specific spinal cord injury cohorts, which should inform preoperative counseling and technique selection [53,54,55].

### 8.4. Optimizing the Seminal Microenvironment

The retrieval of sperm is only the initial stage of a complex therapeutic process, because the seminal plasma in these patients contains a toxic concentration of inflammatory cytokines and reactive oxygen species that can degrade even freshly obtained gametes. To mitigate this post-testicular degradation, clinicians have explored the use of frequent ejaculation protocols. By increasing the frequency of ejaculatory cycles through either repeated stimulation or scheduled retrieval, the residence time of spermatozoa within the hostile environment of the seminal vesicles is reduced [40]. Preliminary data suggest that this flushing effect can lead to a significant improvement in progressive motility and a measurable reduction in the levels of TNF-α and interleukin-6 within the ejaculate. This simple behavioral intervention may effectively reset the local redox balance by preventing the accumulation of aged, apoptotic sperm cells that serve as a primary source of endogenous oxygen radicals, and it can be combined with prompt laboratory processing to limit further oxidative exposure [56,57,58].

## 9. Assisted Reproduction and the Central Role of Sperm DNA Integrity

The recognition that male infertility in spinal cord injury is driven not only by impaired sperm delivery but also by intrinsic sperm dysfunction has important implications for assisted reproduction. Once sperm have been retrieved, success depends not simply on obtaining cells but on the functional and genomic integrity of the cells that are ultimately selected for fertilization [4].

### 9.1. Assisted Reproductive Technologies and Outcomes

Assisted reproductive technologies have significantly expanded the reproductive options available to men with spinal cord injury. Intrauterine insemination may be considered when a sufficient number of motile sperm can be recovered, but in practice the severe asthenozoospermia typical of this population means that in vitro fertilization and, in particular, intracytoplasmic sperm injection (ICSI) are the most commonly used techniques, with ICSI frequently preferred because it can overcome profound deficits in motility and fertilizing capacity [31]. Despite these advances, outcomes remain variable. Fertilization rates with ICSI can be acceptable, but embryo quality, implantation, and live birth are often less predictable [59].

A growing body of evidence indicates that sperm DNA integrity is a central determinant of these outcomes. While ICSI can bypass functional barriers to fertilization, it does not eliminate the impact of underlying genomic instability. Embryos derived from sperm with significant DNA damage may exhibit delayed development, reduced viability, or an increased susceptibility to developmental arrest, and even when implantation occurs the risk of early pregnancy loss appears to be higher [60,61]. Taken together, these findings reinforce the concept that infertility in this population is not merely a problem of sperm delivery or basic semen parameters, but reflects a deeper level of molecular dysfunction that directly influences reproductive success; incorporating DNA fragmentation testing into the diagnostic workup is therefore essential for realistic prognostication and counseling [55]. The principal clinical factors that shape assisted reproductive strategy in this setting are summarized in Table 3.

### 9.2. Laboratory Sperm Processing and Advanced Selection

Given the central role of oxidative stress and inflammation, several laboratory-based strategies have been explored to improve sperm quality prior to fertilization. Conventional sperm processing techniques such as density gradient centrifugation and swim-up are routinely used to isolate motile sperm and reduce exposure to harmful seminal components. While these methods can improve motility and reduce the oxidative burden to some extent, their impact on DNA integrity is more limited, and prolonged or high-force centrifugation may itself generate additional ROS if performed without care [62].

More advanced sperm selection approaches have therefore been proposed, including methods based on membrane integrity, apoptotic marker depletion such as magnetic-activated cell sorting, and hyaluronic acid binding, which enriches for functionally mature spermatozoa. These techniques aim to select sperm with better functional and genomic characteristics, although their specific clinical benefit in spinal cord injury populations requires further validation. Adjunctive strategies, including antioxidant supplementation either in vivo or during in vitro handling, have also been investigated; some studies report improvements in oxidative markers and sperm parameters, but results remain inconsistent and optimal protocols have not been established [63,64]. It is becoming increasingly clear that successful management requires a more individualized approach in which molecular assessments such as sperm DNA fragmentation testing help guide the choice between ejaculated and surgically retrieved sperm and the selection method applied [65,66]. Addressing the biological quality of sperm, alongside effective retrieval methods, thus represents a critical step toward optimizing reproductive success in this population.

## 10. Therapeutic Strategies Targeting Oxidative Stress and Inflammation

The growing recognition that oxidative stress and inflammation are central drivers of infertility in men with spinal cord injury has prompted increasing interest in therapeutic strategies that modify the underlying biological environment, rather than focusing exclusively on retrieval and laboratory manipulation. There is a clear rationale for pharmacological modulation of the reproductive tract, including agents intended to neutralize excess reactive oxygen species, to stabilize the blood–testis barrier and reduce leukocytospermia [67,68], and to enhance sperm motility in vitro, such as pentoxifylline, prior to assisted reproduction. However, translating these mechanistic insights into effective clinical treatments remains a challenge, and current approaches are often heterogeneous in both design and outcome [6,69,70,71,72].

### 10.1. Antioxidant Strategies

Antioxidant therapy represents the most extensively explored intervention, and its rationale is straightforward: by reducing reactive oxygen species and restoring redox balance, it may be possible to protect spermatozoa from ongoing damage and improve functional parameters [67,73]. Commonly studied agents include vitamins C and E, coenzyme Q10, zinc, selenium, carnitines, and folate. These compounds act through complementary mechanisms, including direct scavenging of reactive oxygen species, stabilization of cellular membranes, and support of endogenous antioxidant enzymes such as superoxide dismutase and glutathione peroxidase. In some studies, supplementation has been associated with improvements in sperm motility, viability, and oxidative stress markers.

Unfortunately, the results are not uniform. Variability in dosing, duration, agent combinations, and patient selection makes it difficult to draw definitive conclusions, and there is a theoretical concern that excessive antioxidant supplementation could induce a state of reductive stress that is itself detrimental to the redox-dependent processes of chromatin compaction. In addition, antioxidant therapy may be more effective in preventing damage than in reversing established DNA fragmentation. This distinction is particularly relevant in spinal cord injury, where oxidative stress is chronic and sustained over time, arguing for early and continuous rather than late intervention [74,75].

### 10.2. Anti-Inflammatory and Lifestyle Interventions

Given the close interaction between oxidative stress and inflammation, strategies aimed at modulating the inflammatory response have also been considered. These approaches are less well defined but hold potential, particularly in cases where leukocytospermia and elevated cytokine levels are prominent [76,77,78]. Interventions may include the use of anti-inflammatory agents, although their application in this context remains limited and requires careful consideration due to potential systemic effects. More conservative approaches focus on reducing the sources of inflammation, such as treating subclinical genitourinary infections, optimizing bladder management to reduce the burden of catheter-associated colonization, and minimizing local irritation within the genitourinary tract [79,80].

Lifestyle-related factors should not be overlooked. Physical activity, dietary patterns, weight management, and metabolic health can all influence systemic inflammation and oxidative balance. While these factors are not specific to spinal cord injury, the elevated prevalence of metabolic dysfunction and reduced physical activity after injury makes them especially pertinent, and they represent accessible, low-risk targets for intervention that complement more specific pharmacological measures [81,82,83].

### 10.3. Evidence Base and Clinical Considerations for Antioxidant and Anti-Inflammatory Therapies

The strength of evidence supporting antioxidant and anti-inflammatory interventions in men with spinal cord injury should be interpreted cautiously. Although some antioxidant regimens have been evaluated in randomized or controlled studies of male infertility and have reported improvements in semen parameters or assisted-reproduction outcomes, direct high-quality evidence in the SCI population remains limited. For most agents, including vitamins C and E, coenzyme Q10, zinc, selenium, carnitines, and folate, the available data are derived mainly from heterogeneous studies in broader infertile populations, observational investigations, systematic reviews, or mechanistic and experimental research rather than SCI-specific randomized controlled trials. Consequently, no standardized antioxidant combination, dosage, or treatment duration can currently be recommended specifically for men with SCI. Reported protocols vary considerably in agent selection, dose, combination, and duration, which limits comparison between studies and may partly explain the inconsistent outcomes. Timing is also likely to influence efficacy: early intervention may be more effective in preventing progressive oxidative and mitochondrial injury, whereas treatment initiated after substantial sperm DNA fragmentation has developed may have limited ability to reverse established genomic damage. Anti-inflammatory strategies are supported by an even weaker evidence base and primarily involve treatment of identifiable genitourinary infection, reduction in leukocytospermia, and optimization of bladder management; targeted pharmacological anti-inflammatory therapy remains largely investigational. Therefore, antioxidant or anti-inflammatory treatment should currently be individualized, guided where possible by evidence of oxidative stress or inflammation, and monitored to avoid excessive supplementation and reductive stress.

### 10.4. Emerging and Targeted Approaches

More recent research has begun to explore advanced strategies aimed at delivering antioxidants or anti-inflammatory agents in a more targeted and efficient manner. Nanotechnology-based delivery systems, for example, offer the potential to enhance bioavailability, protect labile compounds from premature degradation, and direct them to specific tissues, although clinical data in this area remain limited [84,85]. Another area of interest is the modulation of the seminal and reproductive tract microenvironment at a molecular level, including investigation into the signaling pathways involved in oxidative stress responses and the role of extracellular vesicles in transferring damaging or protective signals to spermatozoa [86,87]. There is also increasing attention to the potential role of the genitourinary microbiome, as alterations in microbial communities may influence both inflammation and oxidative stress, although this remains an emerging field with many unanswered questions [88]. A simplified overview of current and emerging approaches is presented in Table 4.

Taken together, these therapeutic strategies reflect a shift toward addressing the biological foundations of infertility in spinal cord injury rather than its mechanical consequences alone. While current evidence supports the potential benefit of targeting oxidative stress and inflammation, there is a clear need for more standardized and well-designed studies. Future approaches will likely depend on integrating molecular diagnostics with personalized interventions, allowing treatment to be tailored to the specific oxidative and inflammatory profile of each patient.

## 11. Future Directions

Despite considerable progress in understanding the mechanisms underlying infertility in men with spinal cord injury, several important gaps remain. As a result, translating mechanistic insights into consistent clinical benefit has proven difficult, and a more integrated and methodologically robust approach will be essential [4].

One of the most promising avenues lies in the application of multi-omics technologies. Genomic, transcriptomic, proteomic, and metabolomic analyses have the potential to provide a comprehensive view of the reproductive environment in spinal cord injury. Rather than focusing on isolated markers, these approaches allow the identification of complex molecular signatures that reflect the interaction between oxidative stress, inflammation, and sperm function [4,89,90].

Future research should prioritize the development and validation of standardized biomarker panels capable of characterizing the oxidative and genomic status of spermatozoa in men with spinal cord injury [71,91]. Such panels could combine measurements of seminal reactive oxygen species, total antioxidant capacity, lipid-peroxidation products, inflammatory cytokines, and sperm DNA fragmentation with conventional semen parameters. This approach is supported by evidence linking elevated seminal cytokines and inflammasome activity to impaired sperm motility, as well as by observations that sperm DNA fragmentation is increased in men with spinal cord injury and may influence reproductive outcomes [1]. Such profiles could improve our understanding of disease heterogeneity and help identify subgroups of patients who may benefit from specific interventions.

Future therapeutic studies should also move beyond non-specific antioxidant supplementation by evaluating biomarker-guided combinations, targeted delivery systems, and interventions directed at mitochondrial dysfunction, lipid peroxidation, inflammatory signaling, and the seminal microenvironment. These strategies require carefully standardized dosing and monitoring, since excessive antioxidant exposure may disturb physiological redox signaling [92].

In parallel, assisted reproductive technologies should increasingly integrate sperm DNA fragmentation testing with advanced sperm selection methods, including microfluidic sorting, apoptotic-cell depletion, and selection based on membrane maturity, to enrich spermatozoa with improved functional and genomic integrity. The choice among intrauterine insemination, IVF–ICSI, and surgically retrieved sperm should ultimately be individualized according to semen quality, oxidative burden, DNA integrity, injury characteristics, and the reproductive profile of the couple. Although IVF–ICSI can overcome severe motility impairment, the available evidence indicates that it cannot fully compensate for underlying sperm genomic damage; therefore, prospective multicenter trials should assess fertilization, embryo development, pregnancy, miscarriage, and live birth outcomes rather than improvements in semen parameters alone [3,92].

Longitudinal studies will also be critical. Infertility in spinal cord injury is not a static condition but evolves over time, influenced by factors such as duration of injury, level of neurological impairment, and systemic health. Tracking changes in sperm quality, oxidative stress, and inflammatory markers over extended periods could provide valuable insight into disease progression and treatment response, and would help determine the optimal timing of interventions and of fertility preservation [4,44]. Another important direction involves the development of personalized therapeutic strategies. Given the variability observed among patients, a one-size-fits-all approach is unlikely to be effective; integrating molecular diagnostics with targeted interventions may allow clinicians to tailor treatments based on individual profiles, optimizing outcomes while minimizing unnecessary interventions [93].

Finally, closer collaboration between basic scientists and clinicians will be essential to bridge the gap between experimental findings and real-world applications. Translational research that directly links laboratory observations with clinical outcomes will help ensure that advances in understanding are effectively translated into improved patient care. Taken together, these future directions highlight a shift toward a more precise and individualized model of reproductive medicine in the context of spinal cord injury. By combining advanced molecular tools with thoughtful clinical integration, it may become possible to move beyond symptomatic management and address the underlying biological causes of infertility in this population [94,95,96].

## 12. Conclusions

Male infertility associated with spinal cord injury represents a complex and multifactorial condition that extends well beyond mechanical impairment. While neurogenic dysfunction remains an important component, it does not fully account for the consistently reduced reproductive outcomes observed in affected individuals.

A substantial body of evidence now supports the central role of oxidative stress and inflammation in shaping the seminal environment and impairing sperm function. Elevated reactive oxygen species, reduced antioxidant defenses, and a persistent pro-inflammatory state converge to create conditions that are highly detrimental to spermatozoa. Within this environment, sperm DNA damage and chromatin abnormalities emerge as critical determinants of reproductive success. Importantly, these molecular alterations persist even when sperm are successfully retrieved using advanced techniques, underscoring the limitations of approaches that focus solely on overcoming mechanical barriers. Instead, effective management requires a broader perspective that addresses both the biological quality of sperm and the underlying mechanisms driving dysfunction.

Therapeutic strategies targeting oxidative stress and inflammation show promise, but their clinical application remains limited by variability in the evidence and a lack of standardization. Future progress will depend on the development of reliable biomarkers, improved understanding of disease heterogeneity, and the implementation of personalized treatment approaches. In this context, spinal cord injury-related infertility should be viewed not as a singular condition, but as a dynamic interplay between neural disruption, immune activation, and molecular damage. Recognizing and addressing this complexity is essential for advancing both research and clinical care, with the ultimate goal of improving reproductive outcomes for affected individuals.

## Figures and Tables

**Table 1:** Commonly reported findings about oxidative stress in SCI.

Parameter	SCI Patients	Controls
Reactive oxygen species levels	Increased	Physiological
Total antioxidant capacity	Decreased	Preserved
Lipid peroxidation markers	Elevated	Low
Sperm motility	Reduced	Normal range

**Table 2:** Key clinical associations of sperm DNA damage and chromatin integrity in SCI patients.

Parameter	Observed Effect
Elevated DNA fragmentation index	Reduced fertilization rates
Oxidative DNA lesions	Impaired embryo development
Chromatin immaturity	Lower implantation potential
High DNA damage burden	Increased miscarriage risk

**Table 3:** Clinical factors influencing assisted reproductive strategy in SCI patients.

Factor	Clinical Relevance
Ejaculatory dysfunction	Necessitates assisted sperm retrieval
Reduced sperm motility	Favors intracytoplasmic sperm injection
Elevated DNA fragmentation	Impairs embryo quality
Oxidative stress burden	Reduces overall reproductive success

**Table 4:** A simplified overview of current and emerging approaches.

Strategy	Proposed Effect
Antioxidant supplementation	Reduction in reactive oxygen species
Anti-inflammatory modulation	Decrease in cytokine activity
Advanced sperm selection	Enrichment of functionally competent sperm
Targeted delivery systems	Improved bioavailability of therapeutic agents
Microenvironment modulation	Restoration of seminal balance

## Data Availability

No new data were created or analyzed in this study. Data sharing is not applicable to this article.

## Abbreviations

The following abbreviations are used in this manuscript:

ASAs	Anti-sperm Antibodies
BTB	Blood–Testis Barrier
MMP	Mitochondrial Membrane Potential
ROS	Reactive Oxygen Species
SCI	Spinal Cord Injury
TNF-α	Tumor Necrosis Factor alpha
